# Degradation Rate of 5-Fluorouracil in Metastatic Colorectal Cancer: A New Predictive Outcome Biomarker?

**DOI:** 10.1371/journal.pone.0163105

**Published:** 2016-09-22

**Authors:** Andrea Botticelli, Marina Borro, Concetta Elisa Onesti, Lidia Strigari, Giovanna Gentile, Bruna Cerbelli, Adriana Romiti, Mario Occhipinti, Claudia Sebastiani, Luana Lionetto, Luca Marchetti, Maurizio Simmaco, Paolo Marchetti, Federica Mazzuca

**Affiliations:** 1 Department of Clinical and Molecular Medicine, “Sapienza” University of Rome, Rome, Italy; 2 Department of Neurosciences, Mental Health and Sensory Organs (NESMOS), “Sapienza” University of Rome, Rome, Italy; 3 Medical Oncology Unit, Sant’Andrea Hospital, Rome, Italy; 4 Laboratory of Medical Physics and Expert Systems, Regina Elena National Cancer Institute, Rome, Italy; 5 Istituto Dermopatico dell’Immacolata-IRCCS, Rome, Italy; 6 Department of Radiological Oncological and Pathological Sciences, “Sapienza” University of Rome, Rome, Italy; 7 Department of Medical Oncology, Policlinico Umberto I, Rome, Italy; University of South Alabama Mitchell Cancer Institute, UNITED STATES

## Abstract

**Background:**

5-FU based chemotherapy is the most common first line regimen used for metastatic colorectal cancer (mCRC). Identification of predictive markers of response to chemotherapy is a challenging approach for drug selection. The present study analyzes the predictive role of 5-FU degradation rate (5-FUDR) and genetic polymorphisms (MTHFR, TSER, DPYD) on survival.

**Materials and Methods:**

Genetic polymorphisms of MTHFR, TSER and DPYD, and the 5-FUDR of homogenous patients with mCRC were retrospectively studied. Genetic markers and the 5-FUDR were correlated with clinical outcome.

**Results:**

133 patients affected by mCRC, treated with fluoropyrimidine-based chemotherapy from 2009 to 2014, were evaluated. Patients were classified into three metabolic classes, according to normal distribution of 5-FUDR in more than 1000 patients, as previously published: poor-metabolizer (PM) with 5-FU-DR ≤ 0,85 ng/ml/10^6^ cells/min (8 pts); normal metabolizer with 0,85 < 5-FU-DR < 2,2 ng/ml/10^6^ cells/min (119 pts); ultra-rapid metabolizer (UM) with 5-FU-DR ≥ 2,2 ng/ml/10^6^ cells/min (6 pts). PM and UM groups showed a longer PFS respect to normal metabolizer group (14.5 and 11 months respectively vs 8 months; p = 0.029). A higher G3-4 toxicity rate was observed in PM and UM, respect to normal metabolizer (50% in both PM and UM vs 18%; p = 0.019). No significant associations between genes polymorphisms and outcomes or toxicities were observed.

**Conclusion:**

5-FUDR seems to be significantly involved in predicting survival of patients who underwent 5-FU based CHT for mCRC. Although our findings require confirmation in large prospective studies, they reinforce the concept that individual genetic variation may allow personalized selection of chemotherapy to optimize clinical outcomes.

## Introduction

Colorectal cancer (CRC) is the second highest cause of cancer death in Western countries. The combinations of fluoropyrimidine with oxapliplatin or irinotecan and biological agents are the most common first line chemotherapy regimens used for mCRC. [1–4]

5-Fluorouracil is an antimetabolite of the pyrimidine analogue type, that inhibits DNA and RNA synthesis, with its active metabolites, resulting from anabolism of about 1–2% of the drug. 5-FU active metabolites form an inactive ternary complex with thymidylate synthase (TS) and 5–10-methylenetetrahydrofolate (MTHF). TS optimal inhibition requires an elevated level of MTHF, regulated by the methylenetetrahydrofolate reductase (MTHFR). [5] As a consequence, polymorphisms in TS enhancing region (TSER) and MTHFR gene are presumed to be determinants for 5-FU clinical response, even if their clinical utility is still controversial. [6–14]

Dihydropyrimidine dehydrogenase (DPD) polymorphically expressed enzyme, encoded by DPD gene (DPYD) play a crucial role in the pharmacology of fluoropyrimidines, as it inactivates up to 85% of 5-FU to 5,6-dihydro-5-fluorouracil. [15, 16] Genetic polymorphism in DPYD has shown to be potentially responsible for lethal toxicity after 5FU-based chemotherapy. [17, 18]

Knowledge of the clinical impact of gene polymorphisms involved in the pharmacokinetics and pharmacodynamics of fluoropyrimidines may provide opportunities for patient-tailored chemotherapy, resulting in decreased incidence of severe side effects, reduced numbers of treatment delays or discontinuations, and possibly increased survival probability.

In a previous study high-performance liquid chromatography (HPLC) was used to identify an index of DPD metabolic activity, measuring uracil/dihydrouracil (U/UH2) ratio in plasma. [19] In 2009 we proposed the determination of 5-FU degradation rate (5-FU-DR) by intact peripheral blood monocuclear cells (PBMC) as a useful pre-screening test to evaluate drug toxicity. [20] Furthermore, a genotype-phenotype correlation in 5-FU metabolism was demonstrated, through an association analysis between DPYD single nucleotide polymorphisms (SNPs) and 5-FUDR. [21] Finally, we analysed the effects of the individual 5-FUDR on 5-FU toxicity in a population of 433 CRC patients. We found that both the poor metabolizer (PM) subjects, defined by a 5-FUDR ≤ 5^th^ centile, and the ultra-rapid metabolizer (UM) patients, defined by a 5-FUDR ≥ 95^th^ centile, are at higher risk to develop G3-4 toxicity, with an OR of 3.47 and 3.34, respectively, compared to normal metabolizers (5^th^ < 5FUDR < 95^th^ centiles). [22]

In the present study the Authors aim is to evaluate the influence of genetic polymorphisms of the genes involved in 5-FU metabolism and the of 5-FUDR on progression free survival in a population of metastatic colorectal cancer patients.

## Materials and Methods

### Patients selection

From 2009 to 2014 patients with a histologically confirmed metastatic adenocarcinoma of the colon and rectum undergoing fluoropyrimidine-based chemotherapy at the Sant’Andrea Hospital of Rome, were enrolled in this retrospective study. Each patient records were de-identified and analyzed anonymously.

The inclusions criteria were: patients with measurable disease, adequate organ function and performance status grade 0, 1 or 2 as defined by the Eastern Cooperative Oncology Group; patients who undergone 5-FU and Capecitabine based chemotherapy (FOLFOX, XELOX, FOLFIRI and Capecitabine monotherapy) alone or in combination with biological agents; patients who undergone pre-treatment assay of 5-FUDR and characterization of polymorphisms of TSER, MTHFR and DPYD genes.

The exclusion criteria were: relevant diseases within 6 months (i.e.: myocardial infarction, lung fibrosis, etc); 5FU based chemotherapy in the past.

The study was conducted in accordance with the Declaration of Helsinki and the protocol was approved by the institutional ethic committee.

### Genotyping

Germinal polymorphisms were analyzed. Genomic DNA was isolated from peripheral blood using the X-tractor Gene system (Corbett Life Science, Australia). The splice-site polymorphism, IVS14+1G>A in the *DPYD* gene, C677T and A1298C SNPs in MTHFR gene were analyzed using the commercial kit for fluoropyrimidine response (Diatech, Jesi, Italy) according to manufacturer’s protocol. Briefly, region covering the SNP of interest was amplified by PCR, using specific primers, and then sequenced, using the Pyrosequencer PyroMark ID system (Biotage AB and Biosystems, Uppsala, Sweden). The variable number of tandem repeats (VNTR; 2R or 3R) in TSER was determined by PCR according to manufacturer’s protocol (fluoropyrimidine response—Diatech, Jesi, Italy) and visualized onto 2,2% agarose gel.

#### Determination of the individual 5FU degradation rate

The test was performed using a HPLC-MS/MS instrument including an Agilent 1100 chromatographic system coupled to an API 3200 triple quadrupole (ABSCIEX, Framingham, MA, USA). [15] Briefly, freshly prepared peripheral blood mononuclear cells (2.5–3.5 x 10^6^ cells) are incubate at 37°C, with shaking, with a known amount of 5-FU. Cells aliquots are drawn at time 0, 1 h and 2 h, lysed and centrifuged and the concentration of 5-FU in the supernatants is quantified by HPLC-MS/MS. The 5-FUDR is expressed as ng 5-FU/ml/10^6^ cells/min.

### Chemotherapy response, toxicity and survival

Chemotherapy cycles were administered every 2 or 3 weeks until disease progression or the development of unacceptable toxicity. We focused in patients undergoing chemotherapy consisting of FOLFOX, FOLFIRI, XELOX and Capecitabine with or without BEVACIZUMAB or CETUXIMAB.

Radiological response was assessed with RECIST Criteria. All toxicity was graded according to the National Cancer Institute Common Toxicity Criteria and toxicity assessments performed at day 1 of each cycle until the end of treatment. Patients were also analyzed according to disease control rate (complete response, partial response and stable disease) and progressive disease. Progression-free survival (PFS) was defined as the time from treatment beginning until the first documented tumour progression or death from any cause. Overall survival (OS) was defined as the time from treatment beginning to death from any cause.

### Data analysis and statistics

Patients' data were shown as mean ± SD or median (range) as appropriate. Metabolic classes were determined according to the degradation rates as reported in our previous published article. [22] Box plots of time to progression according the metabolic classes and toxicity were used to show variability among groups. Chi-square test was calculated according to investigated groups.

Patients were also analyzed according to disease control rate (complete response, partial response and stable disease) and progressive disease.

Kaplan-Meier curves were generated according the metabolic classes. Cox multivariate analysis was calculated.

All tests were two-sided, and differences were considered significant at P < 0.05.

All statistics were calculated using R-Package (version 3.1).

## Results

133 metastatic colorectal cancer patients were evaluated in this study. Clinical characteristics and genotype frequencies for MTHFR677/1298, TSER and DPYD are reported in Table 1.

**Table 1:** Baseline characteristics of mCRC patients (n = 133 patients).

	*MEDIAN (RANGE)*	*PATIENTS N*	*%*
***SEX***			
***MALE***		71	53.4%
***FEMALE***		62	46.6%
***AGE***	68 years (32–86)		
***TYPE OF TREATMENT***			
***CAPECITABINE BASED***		20	15.0%
***MONOTHERAPY***		19	95%
***COMBINATION THERAPY***		1	5%
***5-FU BASED***		113	85.0%
***FOLFIRI +/- TARGET AGENT***		60	53.1%
***FOLFOX +/- TARGET AGENT***		53	46.9%
***DPYD***			
***GG***		132	99.2%
***GA***		1	0.8%
***TSER***			
***2R/2R***		36	27.1%
***2R/3R***		57	42.9%
***3R/3R***		40	30.1%
***MTHFR1298***			
***AA***		69	51.9%
***AC***		55	41.4%
***CC***		9	6.8%
***MTHFR677***			
***CC***		37	27.8%
***CT***		65	48.9%
***TT***		31	23.3%
***5-FU-DR (NG/ML/MIN)***	1.610 (0.460–2.570)		
***≤ 0*.*85***		8	6%
***>0*.*85*, *<2*.*2***		119	89%
***≥ 2*.*2***		6	5%

MTHFR677, MTHFR1298 and TSER resulted mutated (heterozygous or homozygous mutated) in 72.2%, 48.2% and 73% of cases, respectively, while DPYD was heterozigously mutated in only one case (0.8%).

The median 5-FUDR value in the overall population was 1.610 (0.460–2.570) ng/ml/10^6^ cells/min.

Overall, 5-FUDR was ≤ 0.85 ng/mL/min in 8 patients (6% of the cases as poor metabolizers, PM) between 0.85 and 2.2 ng/mL/min in 119 patients (89% of the cases as normal metabolizers) and ≥ 2.2 ng/mL/min in 6 patients (5% of the cases as ultra-rapid metabolizers, UM).

### Survival

Information on clinical response to treatment was available for all the 133 patients studied. The median PFS was 8 months, while the median OS was 28 months. No significant associations at the univariate and multivariate analysis were observed between OS and genetic polymorphisms or metabolic classes.

**Fig 1:**
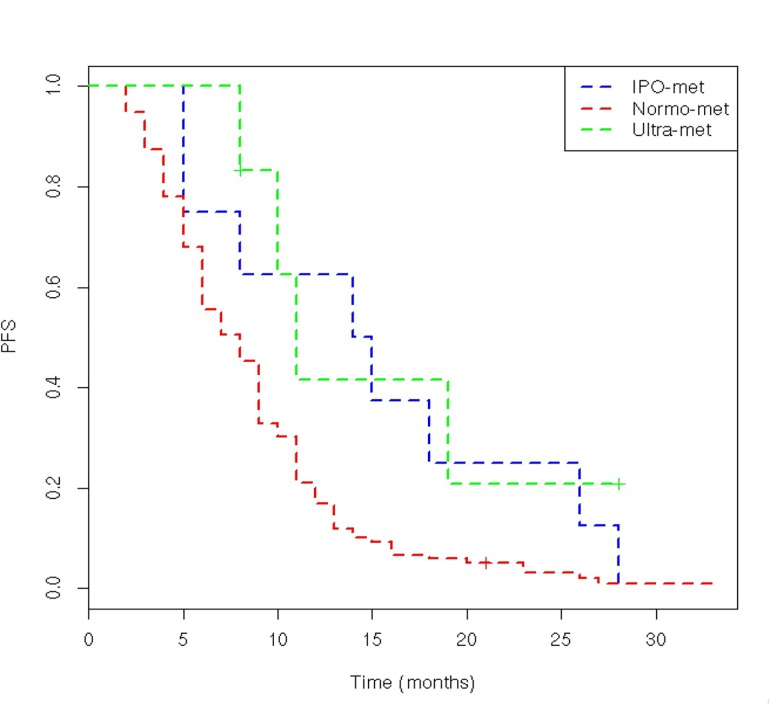
PFS according to the metabolic classes (p = 0.0029).

Patients poor and ultra-rapid metabolizers showed a better median PFS compared to those with normal 5-FUDR (14.5 and 11 vs. 8 months respectively, p = 0.029). (Fig 1; Table 2)

**Table 2:** Progression free survival (PFS) acconding to gene polymorphisms and 5-FU degradation rate (5-FU-DR).

	*MEDIAN PFS (months)*	*PFS RANGE (months)*	*P VALUE*
***ALL PATIENTS***	8.0	2.0–33.0	
***DPYD***			
***GG***	8.0	2.0–33.0	
***GA***	6.0	6.0	NS
***TSER***			
***2R/2R***	7.5	2.0–28.0	
***2R/3R***	8.0	2.0–27.0	
***3R/3R***	9.0	2.0–33.0	NS
***MTHFR1298***			
***AA***	8.0	2.0–28.0	
***AC***	7.0	2.0–33.0	
***CC***	10.0	2.0–20.0	NS
***MTHFR677***			
***CC***	8.0	2.0–28.0	
***CT***	8.0	2.0–33.0	
***TT***	9.0	3.0–28.0	NS
***5-FU-DR (NG/ML/MIN)***			
***≤ 0*.*85***	14.5	5.0–28.0	
***> 0*.*85*, *< 2*.*2***	8.0	2.0–33.0	
***≥ 2*.*2***	11.0	8.0–19.0	0.029

**Fig 2:**
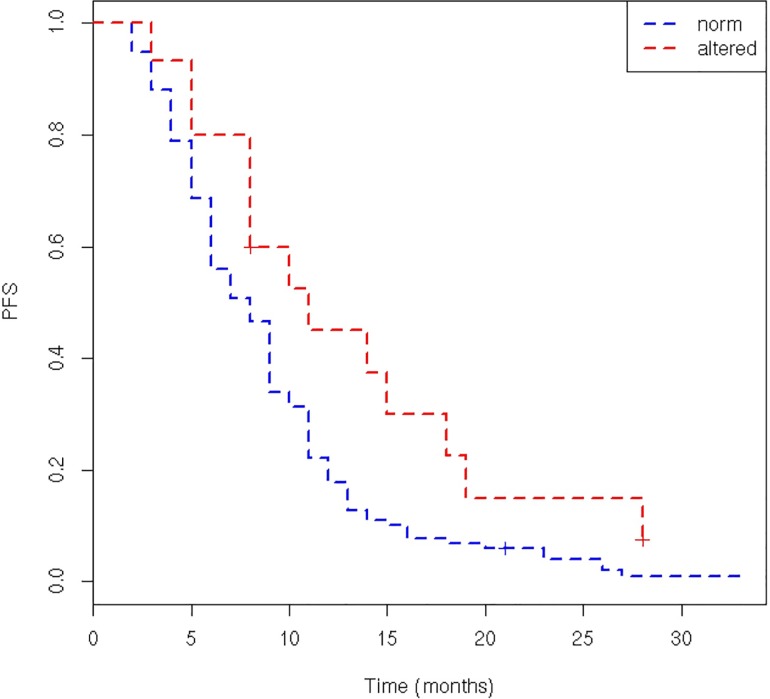
PFS of patients with normal and altered 5-FU-DR (p = 0.03).

PFS of patients with normal 5-FU-DR was lower than PFS of patients with altered 5-FUDR (8 vs 11 months, p = 0.03). (Fig 2)

**Fig 3:**
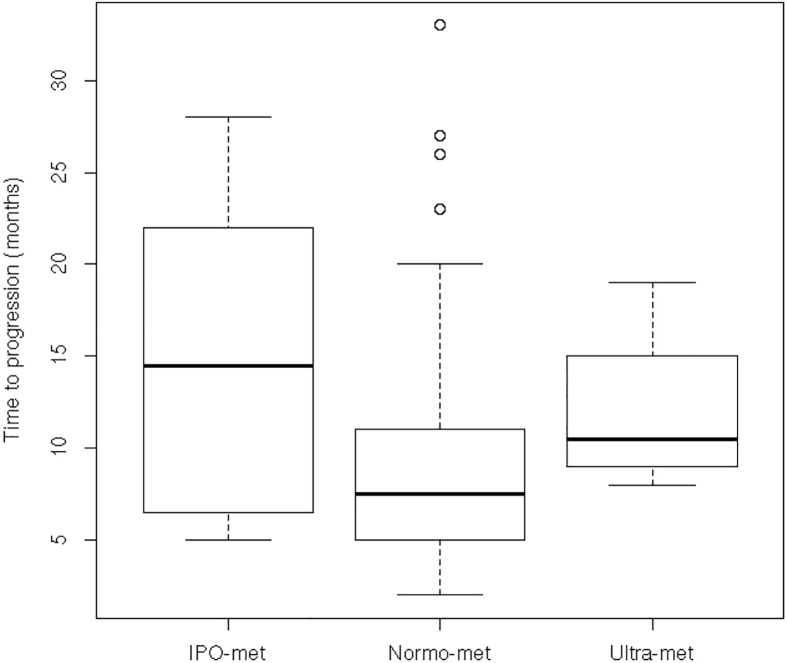
The boxplot of time to progression according to the 5FUDR-based metabolic classes.

The boxplot of time to progression according to the metabolic classes is shown in Fig 3. The median time to progression was higher for patient with a poor than normal and ultra-metabolizer (14.5 vs. 7.5 and 10.5 months respectively).

### Toxicity

Severe toxicities (grade 3 or 4 toxicity) were encountered in 28 patients (21%) and were found to be significantly associated (p = 0.019) with a 5-FUDR below the 5^th^ centile (PM) or above the 95^th^ centile (UM). In particular, the severe toxicity rate was 50% in PM and UM while it was 17.6% in the remaining patients.

In the investigated cohort, 37 patients received a reduced chemotherapy dose. 7 PM subjects received a reduced dose (88.5% of the cases); while in normal and UM groups 23.5% and 33.3% of patients received a reduced dose of 5-FU (p = 0.0004). (Table 3)

**Table 3:** Dose reduction and toxicity according to gene polymorphisms and 5-FU-DR.

	*TOTAL*		*GRADE 0–2*		*GRADE 3–4*		*P VALUE*	*DOSE REDUCTION*		
	N	%	N	%	N	%		N	%	P value
***GENDER***										
***MALE***	71	53.4%	61	85.9%	10	14.1%		19	27.8%	
***FAMALE***	62	46.6%	44	71%	18	29%	NS	17	27.4%	NS
***DPYD***										
***GG***	132	99.2%	104	78.8%	28	21.2%		36	27.3%	
***GA***	1	0.8%	1	100%	0	0%	NS	1	100%	NS
***TSER***										
***2R/2R***	36	27.1%	28	77.8%	8	22.2%		11	30.6%	
***2R/3R***	57	42.9%	45	78.9%	12	21%		17	29.8%	
***3R/3R***	40	30.1%	32	80%	8	20%	NS	9	22.5%	NS
***MTHFR1298***										
***AA***	69	51.9%	52	75.4%	17	24.6%		22	31.9%	
***AC***	55	41.4%	45	81.8%	10	18.2%		10	18.2%	
***CC***	9	6.8%	8	88.9%	1	11.1%	NS	5	55.6%	NS
***MTHFR677***										
***CC***	37	27.8%	32	86.5%	5	13.5%		9	24.3%	
***CT***	65	48.9%	49	75.4%	16	24.6%		17	26.2%	
***TT***	31	23.3%	24	77.4%	7	22.6%	NS	11	35.5%	NS
***5-FU-DR (NG/ML/MIN)***										
***≤ 0*.*85***	8	6%	4	50%	4	50%		7	87.5%	
***>0*.*85*, *<2*.*2***	119	89%	98	82.4%	21	17.6%		28	23.5%	
***≥ 2*.*2***	6	5%	3	50%	3	50%	0.019	2	33.3%	0.0004

## Discussion

The aims of this study was to investigate the efficacy of 5-FU degradation rate and genetic polymorphisms (MTHFR, DPYD, and TSER) as prognostic and predictive parameter for progression free survival.

So far, some studies have investigated the MTHFR, DPYD and TSER genotypes as predictors of toxicity to 5-FU-based chemotherapy. [17, 18, 23–33] The most consistent evidence concerns the DPYD gene, demonstrating an association between severe toxicity and the presence of the polymorphism. [17, 18, 29–33] Interestingly, a case-cohort analysis on the patients enrolled in the phase III CAIRO2 trial showed that DPYD polymorphisms are related to grade 3–4 toxicities, with a trend toward increased overall survival. [17] Moreover, a recent published study, performed on 2038 patients, demonstrated that with a pharmacokinetically guided dose adjustments of 5-FU the incidence and severity of adverse events were significantly reduced, with drug related death decrease from 10% to 0% and G3-4 toxicities risk reduced from 73% to 28%_._ Unfortunately, the authors did not present any results about clinical outcomes, despite 50% dose reduction. [18] Recently, an increasing interest has been shown in identifying prognostic and predictive factor through gene polymorphisms’analysis. Actually, several studies have shown an association between response to treatment and polymorphisms in genes encoding enzymes involved in 5-fluorouracil metabolism, but none of these is considered a prognostic factor in clinical practice. [34–46]

In this study, the distribution of MTHFR677/1298, TSER and DPYD polymorphisms was similar to those described in other Caucasian populations. [47, 48] In our series only one patient presented mutation for DPYD so we could not adequately evaluate the association to efficacy and safety. The effect of genetic polymorphisms of DPYD, TSER, MTHFR and degradation rate of 5-FU on survival was studied. No significant differences in terms of PFS and OS related with MTHFR677/1298, TSER and DPYD polymorphisms were found. These results concur with several other studies that used FOLFOX or FOLFIRI [49–51], but not with tree others that used 5-FU monotherapy and reported better response for patients with the mutated MTHFR677/1298 genotypes [52–54].

Interesting associations with outcomes were found when 5-FUDR was studied. Patients with a low 5-FUDR (≤ 0.85 ng/ml/min) and high 5-FUDR (≥ 2,2 ng/ml/min) presented a significant increase in PFS at the univariate analysis, compared to patients with a normal 5-FUDR. Instead we didn’t find any associations between 5-FUDR and OS, but it could depend that patients lost at follow up reduced the sample size for the analysis of OS.

Moreover, poor metabolizing patients presented a better progression free survival, even though 7 of 8 patients received a reduced dose of 5-FU. This result, enlightens the remarkable finding that probably the pharmacogenetic of these patients allows a longer and effective persistence of 5-FU during treatment.

**Fig 4:**
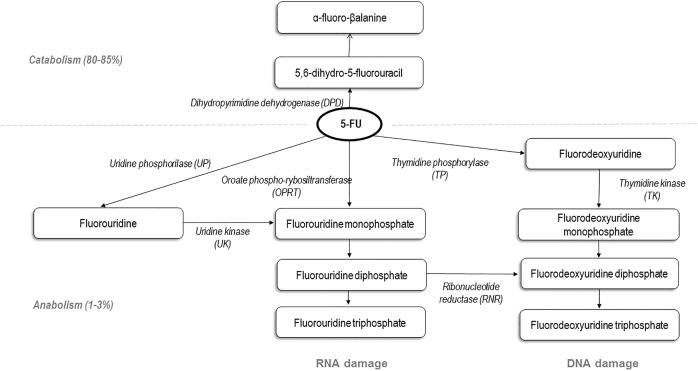
5-fluorouracil metabolism.

Surprisingly, a better outcome and higher toxicity grade (3 patients of 6; 50%) was observed also in UM group. So far a relationship between increased toxicity and/or better outcomes with fast drug metabolism was not reported in literature. The faster 5-FU consumption, expressed with a higher value of 5-FUDR, should be related to an increased DPD activity, with 5-FU inactive metabolites raise, or with an augmented activity of enzymes involved in 5-FU active metabolites production, i.e. orotate phosphoribosyltransferase (ORPT), thymidine phosphorylase (TP) and uridine phosphorilase (UP). [55] In this regard, literature data showed that 5-FU sensitivity is associated with OPRT gene polymorphisms and OPRT/DPD activity ratio. [56–58] This finding leads to the hypothesis that UM show better PFS and higher rate of severe toxicities, due to the increased amount of 5-FU active metabolites. (Fig 4)

The present study focused on the importance of degradation rate of 5-FU and genetic polymorphisms in predicting toxicity and survival of metastatic colorectal cancer patients who underwent 5-FU based chemotherapy. The Authors demonstrated the relevance of 5-FU degradation rate analysis on avoidance of adverse event occurrence, and its role in predicting survival.

Further prospective studies are needed in order to validate and verify these novel and relevant findings. OPRT, UP and TP gene analysis and the dosage of 5-FU metabolites are required to better understand pharmacokinetics mechanisms involved.
